# Methyl 4-(4-chloro­phen­yl)-1,2,3,3a,4,4a,5,12c-octa­hydro­benzo[*f*]chromeno[3,4-*b*]pyrrolizine-4a-carboxyl­ate

**DOI:** 10.1107/S1600536810007804

**Published:** 2010-03-13

**Authors:** B. Gunasekaran, S. Kathiravan, R. Raghunathan, V. Manivannan

**Affiliations:** a Department of Physics, AMET University, Kanathur, Chennai 603 112, India; b Department of Organic Chemistry, University of Madras, Guindy Campus, Chennai 600 025, India; c Department of Research and Development, PRIST University, Vallam, Thanjavur 613 403, Tamil Nadu, India

## Abstract

There are two mol­ecules in the asymmetric unit of the title compound, C_26_H_24_ClNO_3_. The dihedral angles between the naphthalene ring system and the chloro­phenyl substituent are 58.76 (9) and 51.59 (8)° in the two mol­ecules. In the pyrrolizine ring system, both the pyrrolidine rings adopt envelope conformations and the dihydro­pyran rings adopt half-chair conformations. In the pyrrolizine ring system of one of the mol­ecules, one of the C atoms is disordered over two positions with site occupancies of 0.69 (2) and 0.31 (2). The crystal packing is stabilized by weak intra­molecular C—H⋯O inter­actions and the crystal packing is stabilized by weak C—H⋯π inter­actions.

## Related literature

For the biological activity of chromenopyrroles, see: Caine (1993 ▶); Tidey (1992 ▶); Carlson (1993 ▶); Sokoloff *et al.* (1990 ▶); Wilner (1985 ▶). For a related structure, see: Nirmala *et al.* (2009 ▶). For general background to the bridging of N—C bonds in pyrrolizine rings, see: Ramesh *et al.* (2007 ▶). For ring puckering parameters, see: Cremer & Pople (1975 ▶).
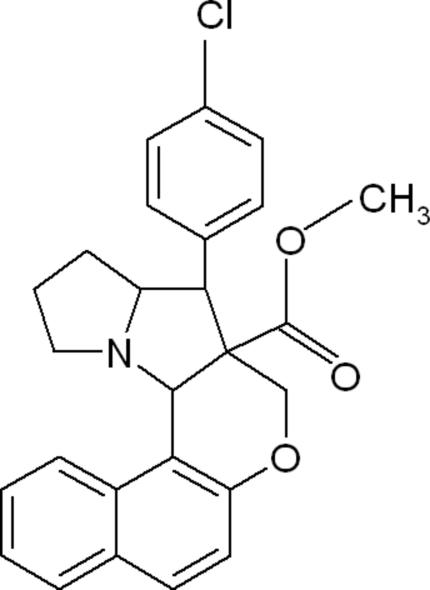


## Experimental

### 

#### Crystal data

C_26_H_24_ClNO_3_
*M* *_r_* = 433.91
Monoclinic, 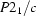

*a* = 22.3214 (9) Å
*b* = 10.8122 (5) Å
*c* = 18.5008 (8) Å
β = 102.756 (3)°
*V* = 4354.8 (3) Å^3^
*Z* = 8
Mo *K*α radiation
μ = 0.20 mm^−1^
*T* = 295 K
0.20 × 0.20 × 0.20 mm

#### Data collection

Bruker Kappa APEXII diffractometer
Absorption correction: multi-scan (*SADABS*; Sheldrick, 1996 ▶) *T*_min_ = 0.960, *T*_max_ = 0.960
40734 measured reflections
10819 independent reflections
5771 reflections with *I* > 2σ(*I*)
*R*_int_ = 0.040

#### Refinement

*R*[*F* ^2^ > 2σ(*F* ^2^)] = 0.063
*wR*(*F* ^2^) = 0.201
*S* = 1.04
10819 reflections
571 parameters
1 restraint
H-atom parameters constrained
Δρ_max_ = 0.64 e Å^−3^
Δρ_min_ = −0.55 e Å^−3^

### 

Data collection: *APEX2* (Bruker, 2004 ▶); cell refinement: *SAINT* (Bruker, 2004 ▶); data reduction: *SAINT*; program(s) used to solve structure: *SHELXS97* (Sheldrick, 2008 ▶); program(s) used to refine structure: *SHELXL97* (Sheldrick, 2008 ▶); molecular graphics: *PLATON* (Spek, 2009 ▶); software used to prepare material for publication: *SHELXL97*.

## Supplementary Material

Crystal structure: contains datablocks global, I. DOI: 10.1107/S1600536810007804/gk2258sup1.cif

Structure factors: contains datablocks I. DOI: 10.1107/S1600536810007804/gk2258Isup2.hkl

Additional supplementary materials:  crystallographic information; 3D view; checkCIF report

## Figures and Tables

**Table 1:** Hydrogen-bond geometry (Å, °) *Cg*5, *Cg*11 and *Cg*12 are the centroids of the C27–C31/C36, C1–C5/C10 and C5–C10 rings, respectively.

*D*—H⋯*A*	*D*—H	H⋯*A*	*D*⋯*A*	*D*—H⋯*A*
C14—H14*A*⋯*Cg*12^i^	0.97	3.00	3.951 (2)	168
C16—H16*A*⋯*Cg*11^i^	0.97	2.83	3.706 (2)	151
C21—H21⋯*Cg*11^ii^	0.93	2.85	3.649 (2)	144
C26—H26*B*⋯*Cg*5^iii^	0.96	2.83	3.555 (1)	133

Symmetry codes: (i) 
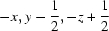
; (ii) 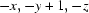; (iii) 
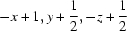
.

**Fig. 1.**
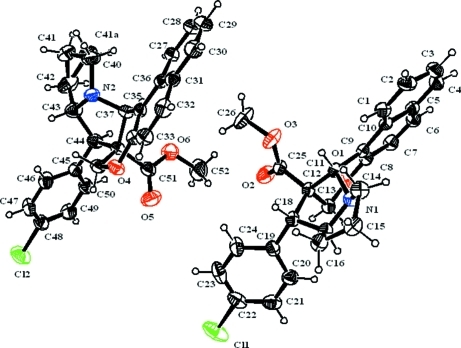
The molecular structure of (I) and (II), with atom labels and 30% probability displacement ellipsoids for non-H atoms.

## supplementary crystallographic information

### Comment

Chromenopyrrole compounds are used in the treatment of impulsive disorders (Caine, 1993), aggressiveness (Tidey, 1992), Parkinson's disease (Carlson, 1993), psychoses, memory disorders (Sokoloff *et al.*, 1990), anxiety and depression (Wilner, 1985).

The geometric parameters of the title molecule (Fig. 1) agree well with reported similar structure (Nirmala *et al.*, 2009). The pyrrolizine ring system is folded about the bridging N1—C17 bond for molecule (I) and N2—C43 bond for molecule (II), as observed in related structures (Ramesh *et al.*, 2007; Nirmala *et al.*, 2009). The dihedral angle between the naphthalene ring system and the chlorophenyl substituent is 58.76 (9) ° for molecule (I) and 51.59 (8) ° for molecule (II).

In the pyrrolizine ring system, both the pyrrolidine rings [N1/C14—C17, N1/C11/C12/C18/C17 for molecule (I) and N2/C40—C43, N2/C37/C38/C44/C43 for molecule (II)] adopt envelope conformations with the puckering parameters (Cremer and Pople, 1975) q2 = 0.3852 (2) Å and φ 2 = 257.36 (2)° and q2 = 0.3627 (2) Å and φ 2 = 71.35 (2)° respectively for molecule (I), q2 = 0.4343 (2) Å and φ 2 = 242.94 (2) ° & q2 = 0.3354 (2) Å and φ 2 = 89.25 (2) ° respectively for molecule (II).

The six-membered heterocyclic ring [C8/C9/C11/C12/C13/O1 for molecule (I) and C34/C35/C37/C38/C39/O4 for molecule (II)] of the benzochromenopyrrole moiety adopts a half-chair conformation with the puckering parameters (Cremer and Pople, 1975) Q = 0.4529 (2) Å, Θ = 132.97 (2) ° and φ = 78.47 (2) ° for molecule (I) and Q = 0.4706 (2) Å, Θ = 132.03 (2) ° and φ = 93.57 (2) ° for molecule (II) respectively. The sum of bond angles around N1 [334.10 (2) °] for molecule (I) and N2 [334.40 (2) °] for molecule (II) indicates the *sp*^3^ hybridization state of atoms N1 & N2.

The molecular structure is stabilized by weak intramolecular C—H···O interactions and the crystal packing is stabilized by weak C—H···π interactions[C14—H14A···Cg12(-X, -1/2+Y, 1/2-Z) distance of 3.951 (2) Å, C16—H16A···Cg11(-X, -1/2+Y, 1/2-Z) distance of 3.706 (2) Å, C21—H21···Cg11(-X, 1-Y, -Z) distance of 3.649 (2) Å and C26—H26B···Cg5 (1-X, 1/2+Y, 1/2-Z) distance of 3.555 (1) Å (Cg5, Cg11 & Cg12 are the centroid of the rings defined by the atoms C27/C28/C29/C30/C31/C36, C1/C2/C3/C4/C5/C10 & C5/C6/C7/C8/C9/C10 respectively)].

### Experimental

A mixture of (*Z*)-methyl 2-((1-formylnaphthalen-2-yloxy) methyl) -3-(4-chloro phenylacrylate (20 mmol) and proline (30 mmol) were refluxed in benzene for 20 h and the solvent was removed under reduced pressure. The crude product was subjected to column chromatography to get the pure product. Chloroform and methanol (1:1) solvent mixture was used for the crystallization by slow evaporation method.

### Refinement

The site occupancy factors of a disordered C atom were refined as C41 = 0.69 (2) and C41A = 0.31 (2) during anisotropic refinement. H atoms were positioned geometrically and refined using riding model with C—H = 0.93Å and *U*_iso_(H) = 1.2Ueq(C) for aromatic, C—H = 0.98Å and *U*_iso_(H) = 1.2Ueq(C) for methine, C—H = 0.97Å and *U*_iso_(H) = 1.2Ueq(C) for methylene , C—H = 0.96Å and *U*_iso_(H) = 1.5Ueq(C) for methyl groups. The components of the anisotropic displacement parameters in direction of the bond of C22 and C23, were restrained to be equal within an effective standard deviation of 0.001 using the DELU command in SHELXL97(Sheldrick, 2008).

### Crystal data

**Table:**

C_26_H_24_ClNO_3_	*F*(000) = 1824
*M**_r_* = 433.91	*D*_x_ = 1.324 Mg m^−^^3^
Monoclinic, *P*2_1_/*c*	Mo *K*α radiation, λ = 0.71073 Å
Hall symbol: -P 2ybc	Cell parameters from 6164 reflections
*a* = 22.3214 (9) Å	θ = 2.5–28.0°
*b* = 10.8122 (5) Å	µ = 0.20 mm^−^^1^
*c* = 18.5008 (8) Å	*T* = 295 K
β = 102.756 (3)°	Block, colourless
*V* = 4354.8 (3) Å^3^	0.20 × 0.20 × 0.20 mm
*Z* = 8	

### Data collection

**Table:**

Bruker Kappa APEXII diffractometer	10819 independent reflections
Radiation source: fine-focus sealed tube	5771 reflections with *I* > 2σ(*I*)
graphite	*R*_int_ = 0.040
Detector resolution: 0 pixels mm^-1^	θ_max_ = 28.3°, θ_min_ = 0.9°
ω and φ scans	*h* = −29→29
Absorption correction: multi-scan (*SADABS*; Sheldrick, 1996)	*k* = −14→14
*T*_min_ = 0.960, *T*_max_ = 0.960	*l* = −24→24
40734 measured reflections	

### Refinement

**Table:**

Refinement on *F*^2^	Primary atom site location: structure-invariant direct methods
Least-squares matrix: full	Secondary atom site location: difference Fourier map
*R*[*F*^2^ > 2σ(*F*^2^)] = 0.063	Hydrogen site location: inferred from neighbouring sites
*wR*(*F*^2^) = 0.201	H-atom parameters constrained
*S* = 1.04	*w* = 1/[σ^2^(*F*_o_^2^) + (0.0823*P*)^2^ + 1.6776*P*] where *P* = (*F*_o_^2^ + 2*F*_c_^2^)/3
10819 reflections	(Δ/σ)_max_ = 0.031
571 parameters	Δρ_max_ = 0.64 e Å^−^^3^
1 restraint	Δρ_min_ = −0.55 e Å^−^^3^

### Fractional atomic coordinates and isotropic or equivalent isotropic displacement parameters (Å^2^)

**Table:**

	*x*	*y*	*z*	*U*_iso_*/*U*_eq_	Occ. (<1)
C1	−0.07636 (12)	0.0026 (3)	0.73870 (14)	0.0533 (6)	
H1	−0.0470	0.0651	0.7470	0.064*	
C2	−0.12729 (14)	0.0129 (3)	0.76812 (16)	0.0641 (8)	
H2	−0.1322	0.0823	0.7960	0.077*	
C3	−0.17171 (14)	−0.0793 (3)	0.75678 (17)	0.0694 (9)	
H3	−0.2068	−0.0701	0.7756	0.083*	
C4	−0.16401 (13)	−0.1825 (3)	0.71836 (16)	0.0631 (8)	
H4	−0.1936	−0.2445	0.7119	0.076*	
C5	−0.11179 (12)	−0.1972 (3)	0.68793 (14)	0.0506 (6)	
C6	−0.10198 (13)	−0.3072 (3)	0.65035 (15)	0.0581 (7)	
H6	−0.1306	−0.3709	0.6452	0.070*	
C7	−0.05150 (12)	−0.3204 (2)	0.62199 (15)	0.0539 (6)	
H7	−0.0449	−0.3940	0.5990	0.065*	
C8	−0.00902 (11)	−0.2231 (2)	0.62717 (13)	0.0455 (6)	
C9	−0.01553 (11)	−0.1132 (2)	0.66223 (12)	0.0431 (5)	
C10	−0.06751 (11)	−0.1020 (2)	0.69571 (12)	0.0450 (6)	
C11	0.03076 (10)	−0.0100 (2)	0.66425 (13)	0.0425 (5)	
H11	0.0413	0.0252	0.7143	0.051*	
C12	0.08965 (11)	−0.0557 (2)	0.64277 (13)	0.0447 (6)	
C13	0.07239 (11)	−0.1440 (2)	0.57766 (13)	0.0474 (6)	
H13A	0.0472	−0.1008	0.5359	0.057*	
H13B	0.1094	−0.1717	0.5632	0.057*	
C14	−0.02061 (14)	0.1973 (2)	0.63721 (16)	0.0601 (7)	
H14A	−0.0045	0.2097	0.6899	0.072*	
H14B	−0.0648	0.1867	0.6283	0.072*	
C15	−0.00443 (14)	0.3051 (3)	0.59322 (16)	0.0606 (7)	
H15A	−0.0323	0.3103	0.5450	0.073*	
H15B	−0.0056	0.3825	0.6194	0.073*	
C16	0.05997 (14)	0.2756 (3)	0.58591 (17)	0.0636 (8)	
H16A	0.0900	0.3021	0.6295	0.076*	
H16B	0.0691	0.3150	0.5425	0.076*	
C17	0.05954 (11)	0.1360 (2)	0.57842 (15)	0.0516 (6)	
H17	0.0511	0.1147	0.5257	0.062*	
C18	0.11705 (11)	0.0657 (3)	0.61847 (14)	0.0507 (6)	
H18	0.1349	0.1120	0.6636	0.061*	
C19	0.16677 (12)	0.0508 (3)	0.57547 (15)	0.0538 (6)	
C20	0.15403 (14)	0.0320 (3)	0.49928 (16)	0.0648 (8)	
H20	0.1133	0.0294	0.4732	0.078*	
C21	0.20021 (16)	0.0171 (3)	0.46145 (19)	0.0745 (9)	
H21	0.1906	0.0033	0.4106	0.089*	
C22	0.25994 (16)	0.0226 (3)	0.4986 (2)	0.0781 (9)	
C23	0.27455 (15)	0.0397 (4)	0.5730 (2)	0.0866 (10)	
H23	0.3155	0.0417	0.5982	0.104*	
C24	0.22811 (13)	0.0542 (3)	0.61148 (19)	0.0744 (9)	
H24	0.2385	0.0665	0.6625	0.089*	
C25	0.13534 (11)	−0.1199 (3)	0.70353 (14)	0.0497 (6)	
C26	0.17634 (18)	−0.1413 (4)	0.83147 (17)	0.0989 (13)	
H26A	0.1757	−0.2298	0.8267	0.148*	
H26B	0.1669	−0.1189	0.8779	0.148*	
H26C	0.2164	−0.1108	0.8297	0.148*	
C27	0.27507 (12)	−0.1797 (3)	1.01938 (14)	0.0554 (7)	
H27	0.2977	−0.2516	1.0183	0.067*	
C28	0.22600 (14)	−0.1821 (3)	1.05207 (16)	0.0714 (9)	
H28	0.2159	−0.2551	1.0731	0.086*	
C29	0.19090 (15)	−0.0768 (4)	1.05429 (18)	0.0810 (10)	
H29	0.1575	−0.0793	1.0767	0.097*	
C30	0.20549 (14)	0.0296 (4)	1.02362 (17)	0.0747 (9)	
H30	0.1822	0.1002	1.0259	0.090*	
C31	0.25532 (12)	0.0358 (3)	0.98824 (14)	0.0561 (7)	
C32	0.26886 (14)	0.1441 (3)	0.95261 (17)	0.0656 (8)	
H32	0.2454	0.2148	0.9538	0.079*	
C33	0.31533 (14)	0.1470 (3)	0.91679 (17)	0.0636 (7)	
H33	0.3230	0.2187	0.8925	0.076*	
C34	0.35220 (12)	0.0419 (3)	0.91605 (14)	0.0513 (6)	
C35	0.34343 (11)	−0.0648 (2)	0.95212 (13)	0.0454 (6)	
C36	0.29225 (11)	−0.0707 (2)	0.98716 (13)	0.0464 (6)	
C37	0.38691 (12)	−0.1712 (2)	0.95372 (14)	0.0503 (6)	
H37	0.3648	−0.2498	0.9517	0.060*	
C38	0.42144 (11)	−0.1641 (3)	0.89047 (14)	0.0515 (6)	
C39	0.44475 (12)	−0.0338 (3)	0.88659 (15)	0.0552 (7)	
H39A	0.4746	−0.0155	0.9320	0.066*	
H39B	0.4655	−0.0279	0.8458	0.066*	
C40	0.42860 (16)	−0.2218 (4)	1.08863 (17)	0.0810 (10)	
H40A	0.3924	−0.2741	1.0787	0.097*	
H40B	0.4230	−0.1575	1.1231	0.097*	
C42	0.5073 (2)	−0.3374 (4)	1.05483 (19)	0.0912 (12)	
H42A	0.4861	−0.4114	1.0331	0.109*	
H42B	0.5512	−0.3533	1.0676	0.109*	
C43	0.49248 (15)	−0.2293 (3)	1.00385 (17)	0.0700 (8)	
H43	0.5272	−0.1716	1.0143	0.084*	
C44	0.47562 (13)	−0.2556 (3)	0.91870 (16)	0.0611 (7)	
H44	0.4591	−0.3398	0.9117	0.073*	
C45	0.52890 (12)	−0.2479 (3)	0.88104 (14)	0.0521 (6)	
C46	0.57228 (13)	−0.1541 (3)	0.89731 (17)	0.0672 (8)	
H46	0.5694	−0.0966	0.9338	0.081*	
C47	0.61934 (13)	−0.1444 (3)	0.86064 (18)	0.0711 (9)	
H47	0.6477	−0.0804	0.8717	0.085*	
C48	0.62386 (13)	−0.2299 (3)	0.80779 (17)	0.0690 (8)	
C49	0.58322 (14)	−0.3254 (3)	0.79190 (17)	0.0719 (9)	
H49	0.5877	−0.3851	0.7572	0.086*	
C50	0.53543 (13)	−0.3326 (3)	0.82786 (16)	0.0633 (8)	
H50	0.5069	−0.3962	0.8159	0.076*	
C51	0.38363 (13)	−0.2008 (3)	0.81484 (17)	0.0628 (7)	
C52	0.3130 (2)	−0.3414 (5)	0.7477 (2)	0.1147 (15)	
H52A	0.2933	−0.2728	0.7191	0.172*	
H52B	0.2824	−0.3982	0.7568	0.172*	
H52C	0.3394	−0.3827	0.7210	0.172*	
N1	0.00836 (9)	0.08961 (18)	0.61034 (11)	0.0453 (5)	
N2	0.43864 (10)	−0.1676 (2)	1.02015 (12)	0.0621 (6)	
O1	0.03955 (8)	−0.24894 (16)	0.59566 (10)	0.0536 (4)	
O2	0.17254 (10)	−0.1925 (2)	0.69264 (11)	0.0794 (7)	
O3	0.13110 (10)	−0.0879 (2)	0.77133 (10)	0.0796 (7)	
O4	0.39666 (9)	0.05623 (18)	0.87646 (11)	0.0625 (5)	
O5	0.38716 (15)	−0.1522 (3)	0.75852 (13)	0.1160 (10)	
O6	0.34906 (11)	−0.2967 (2)	0.81782 (12)	0.0883 (7)	
Cl1	0.31782 (5)	0.00823 (10)	0.44984 (8)	0.1211 (5)	
Cl2	0.68286 (5)	−0.21687 (13)	0.76101 (7)	0.1163 (4)	
C41	0.4847 (5)	−0.2963 (9)	1.1198 (4)	0.077 (2)	0.69 (2)
H41A	0.5153	−0.2462	1.1523	0.092*	0.69 (2)
H41B	0.4746	−0.3666	1.1474	0.092*	0.69 (2)
C41A	0.4516 (12)	−0.3365 (15)	1.0949 (12)	0.080 (5)	0.31 (2)
H41C	0.4206	−0.3954	1.0717	0.096*	0.31 (2)
H41D	0.4656	−0.3585	1.1467	0.096*	0.31 (2)

### Atomic displacement parameters (Å^2^)

**Table:**

	*U*^11^	*U*^22^	*U*^33^	*U*^12^	*U*^13^	*U*^23^
C1	0.0563 (15)	0.0537 (16)	0.0517 (14)	0.0058 (13)	0.0160 (12)	0.0046 (13)
C2	0.0726 (19)	0.0651 (19)	0.0601 (17)	0.0156 (16)	0.0267 (15)	0.0091 (14)
C3	0.0559 (17)	0.087 (2)	0.0709 (19)	0.0122 (17)	0.0256 (15)	0.0190 (18)
C4	0.0485 (15)	0.074 (2)	0.0658 (17)	−0.0056 (14)	0.0099 (13)	0.0191 (16)
C5	0.0484 (14)	0.0522 (16)	0.0482 (13)	−0.0007 (12)	0.0039 (11)	0.0109 (12)
C6	0.0570 (16)	0.0504 (16)	0.0621 (16)	−0.0125 (13)	0.0027 (13)	0.0094 (14)
C7	0.0607 (16)	0.0391 (14)	0.0580 (15)	−0.0042 (12)	0.0046 (13)	−0.0008 (12)
C8	0.0466 (13)	0.0433 (14)	0.0441 (13)	0.0030 (11)	0.0050 (10)	0.0010 (11)
C9	0.0431 (13)	0.0435 (14)	0.0396 (12)	0.0019 (11)	0.0022 (10)	0.0007 (10)
C10	0.0467 (13)	0.0461 (14)	0.0397 (12)	0.0045 (11)	0.0041 (10)	0.0065 (11)
C11	0.0430 (12)	0.0414 (13)	0.0401 (12)	0.0009 (11)	0.0028 (10)	−0.0042 (10)
C12	0.0404 (12)	0.0469 (14)	0.0452 (13)	0.0007 (11)	0.0056 (10)	−0.0050 (11)
C13	0.0450 (13)	0.0482 (14)	0.0489 (13)	0.0010 (11)	0.0102 (11)	−0.0073 (12)
C14	0.0701 (18)	0.0437 (15)	0.0685 (17)	0.0104 (14)	0.0196 (14)	−0.0036 (13)
C15	0.078 (2)	0.0399 (14)	0.0595 (16)	0.0069 (14)	0.0053 (14)	−0.0064 (13)
C16	0.0706 (19)	0.0463 (16)	0.0682 (18)	−0.0064 (14)	0.0032 (15)	0.0058 (14)
C17	0.0484 (14)	0.0508 (15)	0.0532 (14)	−0.0007 (12)	0.0062 (12)	−0.0016 (12)
C18	0.0444 (13)	0.0535 (16)	0.0518 (14)	−0.0036 (12)	0.0053 (11)	−0.0051 (12)
C19	0.0455 (14)	0.0514 (16)	0.0641 (17)	−0.0019 (12)	0.0113 (12)	0.0039 (13)
C20	0.0602 (17)	0.073 (2)	0.0630 (18)	0.0105 (15)	0.0176 (14)	0.0129 (15)
C21	0.084 (2)	0.072 (2)	0.076 (2)	0.0168 (18)	0.0353 (18)	0.0229 (17)
C22	0.072 (2)	0.0567 (19)	0.119 (2)	0.0069 (16)	0.050 (2)	0.0200 (19)
C23	0.0443 (17)	0.088 (3)	0.127 (2)	−0.0065 (17)	0.0175 (19)	−0.003 (2)
C24	0.0461 (16)	0.088 (2)	0.085 (2)	−0.0089 (16)	0.0061 (15)	−0.0102 (19)
C25	0.0447 (14)	0.0540 (16)	0.0474 (14)	0.0028 (12)	0.0036 (11)	−0.0038 (12)
C26	0.108 (3)	0.115 (3)	0.0555 (18)	0.041 (3)	−0.0202 (19)	−0.007 (2)
C27	0.0509 (15)	0.0674 (18)	0.0502 (14)	−0.0046 (13)	0.0160 (12)	−0.0017 (13)
C28	0.0662 (19)	0.091 (2)	0.0635 (18)	−0.0082 (18)	0.0277 (15)	0.0031 (17)
C29	0.070 (2)	0.116 (3)	0.068 (2)	0.002 (2)	0.0381 (17)	−0.001 (2)
C30	0.0666 (19)	0.094 (3)	0.0678 (19)	0.0193 (18)	0.0241 (16)	−0.0140 (19)
C31	0.0545 (16)	0.0656 (19)	0.0474 (14)	0.0027 (14)	0.0093 (12)	−0.0116 (13)
C32	0.0655 (18)	0.0573 (18)	0.0724 (19)	0.0120 (15)	0.0122 (15)	−0.0107 (15)
C33	0.0723 (19)	0.0487 (16)	0.0681 (18)	0.0009 (15)	0.0118 (15)	0.0030 (14)
C34	0.0491 (14)	0.0536 (16)	0.0511 (14)	−0.0029 (12)	0.0107 (12)	0.0007 (12)
C35	0.0436 (13)	0.0488 (14)	0.0435 (13)	−0.0030 (11)	0.0091 (10)	−0.0038 (11)
C36	0.0441 (13)	0.0550 (16)	0.0396 (12)	−0.0003 (12)	0.0079 (10)	−0.0060 (11)
C37	0.0530 (15)	0.0497 (15)	0.0529 (14)	−0.0006 (12)	0.0218 (12)	0.0035 (12)
C38	0.0482 (14)	0.0588 (17)	0.0509 (14)	−0.0023 (13)	0.0182 (12)	0.0007 (12)
C39	0.0483 (14)	0.0633 (18)	0.0562 (15)	0.0002 (13)	0.0165 (12)	0.0087 (13)
C40	0.080 (2)	0.112 (3)	0.0537 (17)	0.014 (2)	0.0214 (16)	0.0244 (19)
C42	0.119 (3)	0.076 (2)	0.070 (2)	0.036 (2)	0.005 (2)	0.0069 (18)
C43	0.073 (2)	0.078 (2)	0.0632 (17)	0.0127 (17)	0.0224 (15)	0.0039 (16)
C44	0.0578 (16)	0.0643 (18)	0.0666 (17)	0.0092 (14)	0.0251 (14)	0.0030 (15)
C45	0.0490 (14)	0.0588 (16)	0.0496 (14)	0.0066 (13)	0.0134 (11)	−0.0037 (13)
C46	0.0606 (17)	0.075 (2)	0.0661 (18)	−0.0013 (16)	0.0133 (14)	−0.0255 (16)
C47	0.0482 (16)	0.078 (2)	0.086 (2)	−0.0123 (15)	0.0132 (15)	−0.0168 (18)
C48	0.0539 (17)	0.089 (2)	0.0698 (19)	−0.0023 (17)	0.0254 (14)	−0.0062 (18)
C49	0.074 (2)	0.083 (2)	0.0637 (18)	−0.0030 (18)	0.0258 (16)	−0.0267 (17)
C50	0.0580 (16)	0.0649 (19)	0.0680 (18)	−0.0074 (14)	0.0159 (14)	−0.0148 (15)
C51	0.0601 (17)	0.073 (2)	0.0603 (17)	−0.0082 (15)	0.0238 (14)	−0.0043 (16)
C52	0.109 (3)	0.126 (4)	0.105 (3)	−0.038 (3)	0.014 (3)	−0.046 (3)
N1	0.0470 (11)	0.0398 (11)	0.0488 (11)	0.0032 (9)	0.0102 (9)	−0.0035 (9)
N2	0.0550 (13)	0.0814 (18)	0.0520 (12)	0.0137 (12)	0.0163 (11)	0.0146 (12)
O1	0.0583 (11)	0.0422 (10)	0.0610 (11)	0.0004 (8)	0.0149 (9)	−0.0101 (8)
O2	0.0765 (14)	0.0942 (17)	0.0653 (12)	0.0387 (13)	0.0108 (11)	0.0031 (12)
O3	0.0849 (15)	0.0937 (17)	0.0486 (11)	0.0366 (13)	−0.0099 (10)	−0.0138 (11)
O4	0.0589 (11)	0.0575 (12)	0.0757 (13)	0.0010 (9)	0.0248 (10)	0.0177 (10)
O5	0.150 (3)	0.135 (3)	0.0572 (14)	−0.053 (2)	0.0100 (15)	0.0066 (15)
O6	0.1044 (18)	0.0896 (17)	0.0706 (14)	−0.0384 (15)	0.0188 (13)	−0.0166 (13)
Cl1	0.1133 (8)	0.0878 (7)	0.1952 (13)	0.0268 (6)	0.1052 (9)	0.0392 (7)
Cl2	0.0892 (7)	0.1540 (11)	0.1256 (9)	−0.0129 (7)	0.0664 (6)	−0.0095 (8)
C41	0.096 (6)	0.072 (4)	0.057 (3)	0.006 (4)	0.007 (3)	0.005 (3)
C41A	0.109 (13)	0.061 (8)	0.066 (9)	−0.010 (8)	0.009 (9)	0.023 (6)

### Geometric parameters (Å, °)

**Table:**

C1—C2	1.369 (4)	C27—H27	0.9300
C1—C10	1.421 (4)	C28—C29	1.388 (5)
C1—H1	0.9300	C28—H28	0.9300
C2—C3	1.389 (4)	C29—C30	1.354 (5)
C2—H2	0.9300	C29—H29	0.9300
C3—C4	1.354 (4)	C30—C31	1.411 (4)
C3—H3	0.9300	C30—H30	0.9300
C4—C5	1.411 (4)	C31—C32	1.409 (4)
C4—H4	0.9300	C31—C36	1.419 (4)
C5—C10	1.412 (4)	C32—C33	1.349 (4)
C5—C6	1.418 (4)	C32—H32	0.9300
C6—C7	1.351 (4)	C33—C34	1.405 (4)
C6—H6	0.9300	C33—H33	0.9300
C7—C8	1.405 (4)	C34—O4	1.366 (3)
C7—H7	0.9300	C34—C35	1.369 (4)
C8—O1	1.368 (3)	C35—C36	1.434 (3)
C8—C9	1.376 (3)	C35—C37	1.501 (4)
C9—C10	1.436 (3)	C37—N2	1.489 (4)
C9—C11	1.516 (3)	C37—C38	1.539 (3)
C11—N1	1.478 (3)	C37—H37	0.9800
C11—C12	1.536 (3)	C38—C39	1.509 (4)
C11—H11	0.9800	C38—C51	1.518 (4)
C12—C25	1.510 (3)	C38—C44	1.561 (4)
C12—C13	1.519 (3)	C39—O4	1.430 (3)
C12—C18	1.555 (4)	C39—H39A	0.9700
C13—O1	1.429 (3)	C39—H39B	0.9700
C13—H13A	0.9700	C40—C41A	1.338 (14)
C13—H13B	0.9700	C40—N2	1.457 (4)
C14—N1	1.471 (3)	C40—C41	1.493 (8)
C14—C15	1.510 (4)	C40—H40A	0.9700
C14—H14A	0.9700	C40—H40B	0.9700
C14—H14B	0.9700	C42—C41	1.471 (7)
C15—C16	1.507 (4)	C42—C43	1.492 (5)
C15—H15A	0.9700	C42—C41A	1.582 (17)
C15—H15B	0.9700	C42—H42A	0.9700
C16—C17	1.516 (4)	C42—H42B	0.9700
C16—H16A	0.9700	C43—N2	1.463 (4)
C16—H16B	0.9700	C43—C44	1.563 (4)
C17—N1	1.484 (3)	C43—H43	0.9800
C17—C18	1.535 (4)	C44—C45	1.507 (4)
C17—H17	0.9800	C44—H44	0.9800
C18—C19	1.510 (3)	C45—C50	1.375 (4)
C18—H18	0.9800	C45—C46	1.388 (4)
C19—C24	1.385 (4)	C46—C47	1.375 (4)
C19—C20	1.390 (4)	C46—H46	0.9300
C20—C21	1.377 (4)	C47—C48	1.365 (4)
C20—H20	0.9300	C47—H47	0.9300
C21—C22	1.360 (5)	C48—C49	1.364 (4)
C21—H21	0.9300	C48—Cl2	1.734 (3)
C22—C23	1.355 (5)	C49—C50	1.378 (4)
C22—Cl1	1.739 (3)	C49—H49	0.9300
C23—C24	1.390 (4)	C50—H50	0.9300
C23—H23	0.9300	C51—O5	1.185 (3)
C24—H24	0.9300	C51—O6	1.301 (4)
C25—O2	1.192 (3)	C52—O6	1.450 (4)
C25—O3	1.325 (3)	C52—H52A	0.9600
C26—O3	1.448 (4)	C52—H52B	0.9600
C26—H26A	0.9600	C52—H52C	0.9600
C26—H26B	0.9600	C41—H41A	0.9700
C26—H26C	0.9600	C41—H41B	0.9700
C27—C28	1.363 (4)	C41A—H41C	0.9700
C27—C36	1.411 (4)	C41A—H41D	0.9700
			
C2—C1—C10	121.2 (3)	C32—C31—C30	121.9 (3)
C2—C1—H1	119.4	C32—C31—C36	118.9 (2)
C10—C1—H1	119.4	C30—C31—C36	119.2 (3)
C1—C2—C3	120.6 (3)	C33—C32—C31	121.1 (3)
C1—C2—H2	119.7	C33—C32—H32	119.4
C3—C2—H2	119.7	C31—C32—H32	119.4
C4—C3—C2	120.1 (3)	C32—C33—C34	120.1 (3)
C4—C3—H3	119.9	C32—C33—H33	120.0
C2—C3—H3	119.9	C34—C33—H33	120.0
C3—C4—C5	121.0 (3)	O4—C34—C35	123.8 (2)
C3—C4—H4	119.5	O4—C34—C33	114.2 (2)
C5—C4—H4	119.5	C35—C34—C33	122.0 (2)
C4—C5—C10	119.9 (3)	C34—C35—C36	118.2 (2)
C4—C5—C6	121.4 (3)	C34—C35—C37	119.6 (2)
C10—C5—C6	118.7 (2)	C36—C35—C37	122.2 (2)
C7—C6—C5	120.8 (3)	C27—C36—C31	117.3 (2)
C7—C6—H6	119.6	C27—C36—C35	123.1 (2)
C5—C6—H6	119.6	C31—C36—C35	119.6 (2)
C6—C7—C8	120.1 (3)	N2—C37—C35	112.1 (2)
C6—C7—H7	119.9	N2—C37—C38	101.48 (19)
C8—C7—H7	119.9	C35—C37—C38	112.2 (2)
O1—C8—C9	123.6 (2)	N2—C37—H37	110.3
O1—C8—C7	113.8 (2)	C35—C37—H37	110.3
C9—C8—C7	122.6 (2)	C38—C37—H37	110.3
C8—C9—C10	117.1 (2)	C39—C38—C51	109.1 (2)
C8—C9—C11	120.3 (2)	C39—C38—C37	108.6 (2)
C10—C9—C11	122.6 (2)	C51—C38—C37	114.7 (2)
C5—C10—C1	117.2 (2)	C39—C38—C44	111.0 (2)
C5—C10—C9	120.6 (2)	C51—C38—C44	112.0 (2)
C1—C10—C9	122.3 (2)	C37—C38—C44	101.3 (2)
N1—C11—C9	113.13 (18)	O4—C39—C38	112.6 (2)
N1—C11—C12	103.94 (18)	O4—C39—H39A	109.1
C9—C11—C12	112.0 (2)	C38—C39—H39A	109.1
N1—C11—H11	109.2	O4—C39—H39B	109.1
C9—C11—H11	109.2	C38—C39—H39B	109.1
C12—C11—H11	109.2	H39A—C39—H39B	107.8
C25—C12—C13	108.2 (2)	C41A—C40—N2	108.5 (6)
C25—C12—C11	115.5 (2)	C41A—C40—C41	36.2 (9)
C13—C12—C11	109.01 (19)	N2—C40—C41	106.0 (3)
C25—C12—C18	111.1 (2)	C41A—C40—H40A	76.4
C13—C12—C18	110.4 (2)	N2—C40—H40A	110.5
C11—C12—C18	102.53 (19)	C41—C40—H40A	110.5
O1—C13—C12	111.57 (19)	C41A—C40—H40B	135.2
O1—C13—H13A	109.3	N2—C40—H40B	110.5
C12—C13—H13A	109.3	C41—C40—H40B	110.5
O1—C13—H13B	109.3	H40A—C40—H40B	108.7
C12—C13—H13B	109.3	C41—C42—C43	102.2 (3)
H13A—C13—H13B	108.0	C41—C42—C41A	33.7 (6)
N1—C14—C15	104.7 (2)	C43—C42—C41A	101.7 (5)
N1—C14—H14A	110.8	C41—C42—H42A	111.3
C15—C14—H14A	110.8	C43—C42—H42A	111.3
N1—C14—H14B	110.8	C41A—C42—H42A	80.7
C15—C14—H14B	110.8	C41—C42—H42B	111.3
H14A—C14—H14B	108.9	C43—C42—H42B	111.3
C16—C15—C14	103.4 (2)	C41A—C42—H42B	137.9
C16—C15—H15A	111.1	H42A—C42—H42B	109.2
C14—C15—H15A	111.1	N2—C43—C42	107.7 (3)
C16—C15—H15B	111.1	N2—C43—C44	105.3 (2)
C14—C15—H15B	111.1	C42—C43—C44	117.7 (3)
H15A—C15—H15B	109.1	N2—C43—H43	108.6
C15—C16—C17	103.4 (2)	C42—C43—H43	108.6
C15—C16—H16A	111.1	C44—C43—H43	108.6
C17—C16—H16A	111.1	C45—C44—C38	116.3 (2)
C15—C16—H16B	111.1	C45—C44—C43	114.6 (2)
C17—C16—H16B	111.1	C38—C44—C43	102.6 (2)
H16A—C16—H16B	109.0	C45—C44—H44	107.6
N1—C17—C16	106.9 (2)	C38—C44—H44	107.6
N1—C17—C18	106.0 (2)	C43—C44—H44	107.6
C16—C17—C18	117.5 (2)	C50—C45—C46	117.5 (2)
N1—C17—H17	108.7	C50—C45—C44	120.9 (3)
C16—C17—H17	108.7	C46—C45—C44	121.6 (2)
C18—C17—H17	108.7	C47—C46—C45	121.4 (3)
C19—C18—C17	115.6 (2)	C47—C46—H46	119.3
C19—C18—C12	116.4 (2)	C45—C46—H46	119.3
C17—C18—C12	102.54 (19)	C48—C47—C46	119.2 (3)
C19—C18—H18	107.3	C48—C47—H47	120.4
C17—C18—H18	107.3	C46—C47—H47	120.4
C12—C18—H18	107.3	C49—C48—C47	121.1 (3)
C24—C19—C20	116.8 (3)	C49—C48—Cl2	119.8 (2)
C24—C19—C18	120.5 (3)	C47—C48—Cl2	119.2 (3)
C20—C19—C18	122.7 (2)	C48—C49—C50	119.2 (3)
C21—C20—C19	121.6 (3)	C48—C49—H49	120.4
C21—C20—H20	119.2	C50—C49—H49	120.4
C19—C20—H20	119.2	C45—C50—C49	121.6 (3)
C22—C21—C20	119.9 (3)	C45—C50—H50	119.2
C22—C21—H21	120.1	C49—C50—H50	119.2
C20—C21—H21	120.1	O5—C51—O6	123.1 (3)
C23—C22—C21	120.6 (3)	O5—C51—C38	124.3 (3)
C23—C22—Cl1	120.0 (3)	O6—C51—C38	112.5 (3)
C21—C22—Cl1	119.4 (3)	O6—C52—H52A	109.5
C22—C23—C24	119.7 (3)	O6—C52—H52B	109.5
C22—C23—H23	120.1	H52A—C52—H52B	109.5
C24—C23—H23	120.1	O6—C52—H52C	109.5
C19—C24—C23	121.4 (3)	H52A—C52—H52C	109.5
C19—C24—H24	119.3	H52B—C52—H52C	109.5
C23—C24—H24	119.3	C14—N1—C11	117.21 (19)
O2—C25—O3	122.1 (2)	C14—N1—C17	107.5 (2)
O2—C25—C12	124.0 (2)	C11—N1—C17	109.84 (18)
O3—C25—C12	114.0 (2)	C40—N2—C43	106.7 (2)
O3—C26—H26A	109.5	C40—N2—C37	117.8 (2)
O3—C26—H26B	109.5	C43—N2—C37	109.9 (2)
H26A—C26—H26B	109.5	C8—O1—C13	115.63 (19)
O3—C26—H26C	109.5	C25—O3—C26	116.1 (2)
H26A—C26—H26C	109.5	C34—O4—C39	117.8 (2)
H26B—C26—H26C	109.5	C51—O6—C52	116.4 (3)
C28—C27—C36	121.5 (3)	C42—C41—C40	105.0 (5)
C28—C27—H27	119.2	C42—C41—H41A	110.8
C36—C27—H27	119.2	C40—C41—H41A	110.8
C27—C28—C29	120.8 (3)	C42—C41—H41B	110.8
C27—C28—H28	119.6	C40—C41—H41B	110.8
C29—C28—H28	119.6	H41A—C41—H41B	108.8
C30—C29—C28	119.7 (3)	C40—C41A—C42	107.0 (10)
C30—C29—H29	120.2	C40—C41A—H41C	110.3
C28—C29—H29	120.2	C42—C41A—H41C	110.3
C29—C30—C31	121.4 (3)	C40—C41A—H41D	110.3
C29—C30—H30	119.3	C42—C41A—H41D	110.3
C31—C30—H30	119.3	H41C—C41A—H41D	108.6
			
C10—C1—C2—C3	0.2 (4)	C34—C35—C36—C27	173.4 (2)
C1—C2—C3—C4	2.1 (5)	C37—C35—C36—C27	−6.4 (4)
C2—C3—C4—C5	−1.4 (4)	C34—C35—C36—C31	−4.6 (3)
C3—C4—C5—C10	−1.7 (4)	C37—C35—C36—C31	175.6 (2)
C3—C4—C5—C6	177.3 (3)	C34—C35—C37—N2	91.9 (3)
C4—C5—C6—C7	−179.5 (2)	C36—C35—C37—N2	−88.3 (3)
C10—C5—C6—C7	−0.5 (4)	C34—C35—C37—C38	−21.6 (3)
C5—C6—C7—C8	−2.3 (4)	C36—C35—C37—C38	158.2 (2)
C6—C7—C8—O1	−179.5 (2)	N2—C37—C38—C39	−73.7 (3)
C6—C7—C8—C9	1.7 (4)	C35—C37—C38—C39	46.2 (3)
O1—C8—C9—C10	−177.1 (2)	N2—C37—C38—C51	164.0 (2)
C7—C8—C9—C10	1.5 (3)	C35—C37—C38—C51	−76.2 (3)
O1—C8—C9—C11	3.2 (4)	N2—C37—C38—C44	43.2 (3)
C7—C8—C9—C11	−178.2 (2)	C35—C37—C38—C44	163.0 (2)
C4—C5—C10—C1	3.9 (3)	C51—C38—C39—O4	68.7 (3)
C6—C5—C10—C1	−175.1 (2)	C37—C38—C39—O4	−57.0 (3)
C4—C5—C10—C9	−177.2 (2)	C44—C38—C39—O4	−167.5 (2)
C6—C5—C10—C9	3.8 (3)	C41—C42—C43—N2	−28.8 (7)
C2—C1—C10—C5	−3.2 (4)	C41A—C42—C43—N2	5.7 (11)
C2—C1—C10—C9	177.9 (2)	C41—C42—C43—C44	−147.6 (6)
C8—C9—C10—C5	−4.3 (3)	C41A—C42—C43—C44	−113.1 (11)
C11—C9—C10—C5	175.5 (2)	C39—C38—C44—C45	−49.2 (3)
C8—C9—C10—C1	174.6 (2)	C51—C38—C44—C45	73.0 (3)
C11—C9—C10—C1	−5.6 (3)	C37—C38—C44—C45	−164.3 (2)
C8—C9—C11—N1	102.2 (2)	C39—C38—C44—C43	76.8 (3)
C10—C9—C11—N1	−77.5 (3)	C51—C38—C44—C43	−161.0 (2)
C8—C9—C11—C12	−14.9 (3)	C37—C38—C44—C43	−38.3 (3)
C10—C9—C11—C12	165.4 (2)	N2—C43—C44—C45	146.1 (3)
N1—C11—C12—C25	157.0 (2)	C42—C43—C44—C45	−93.8 (4)
C9—C11—C12—C25	−80.6 (3)	N2—C43—C44—C38	19.1 (3)
N1—C11—C12—C13	−80.9 (2)	C42—C43—C44—C38	139.2 (3)
C9—C11—C12—C13	41.5 (3)	C38—C44—C45—C50	−101.4 (3)
N1—C11—C12—C18	36.0 (2)	C43—C44—C45—C50	139.0 (3)
C9—C11—C12—C18	158.50 (19)	C38—C44—C45—C46	77.1 (4)
C25—C12—C13—O1	66.2 (2)	C43—C44—C45—C46	−42.5 (4)
C11—C12—C13—O1	−60.2 (3)	C50—C45—C46—C47	1.3 (5)
C18—C12—C13—O1	−172.10 (19)	C44—C45—C46—C47	−177.2 (3)
N1—C14—C15—C16	−36.6 (3)	C45—C46—C47—C48	−0.9 (5)
C14—C15—C16—C17	35.5 (3)	C46—C47—C48—C49	−1.2 (5)
C15—C16—C17—N1	−21.8 (3)	C46—C47—C48—Cl2	179.5 (3)
C15—C16—C17—C18	−140.6 (2)	C47—C48—C49—C50	2.6 (5)
N1—C17—C18—C19	153.3 (2)	Cl2—C48—C49—C50	−178.0 (3)
C16—C17—C18—C19	−87.4 (3)	C46—C45—C50—C49	0.2 (4)
N1—C17—C18—C12	25.6 (2)	C44—C45—C50—C49	178.7 (3)
C16—C17—C18—C12	144.9 (2)	C48—C49—C50—C45	−2.1 (5)
C25—C12—C18—C19	71.1 (3)	C39—C38—C51—O5	20.7 (4)
C13—C12—C18—C19	−48.9 (3)	C37—C38—C51—O5	142.7 (3)
C11—C12—C18—C19	−164.9 (2)	C44—C38—C51—O5	−102.6 (4)
C25—C12—C18—C17	−161.7 (2)	C39—C38—C51—O6	−163.0 (2)
C13—C12—C18—C17	78.3 (2)	C37—C38—C51—O6	−41.0 (4)
C11—C12—C18—C17	−37.7 (2)	C44—C38—C51—O6	73.7 (3)
C17—C18—C19—C24	144.5 (3)	C15—C14—N1—C11	147.4 (2)
C12—C18—C19—C24	−95.1 (3)	C15—C14—N1—C17	23.1 (3)
C17—C18—C19—C20	−36.2 (4)	C9—C11—N1—C14	94.5 (3)
C12—C18—C19—C20	84.2 (3)	C12—C11—N1—C14	−143.8 (2)
C24—C19—C20—C21	0.2 (5)	C9—C11—N1—C17	−142.5 (2)
C18—C19—C20—C21	−179.2 (3)	C12—C11—N1—C17	−20.8 (2)
C19—C20—C21—C22	−1.0 (5)	C16—C17—N1—C14	−0.8 (3)
C20—C21—C22—C23	1.6 (5)	C18—C17—N1—C14	125.3 (2)
C20—C21—C22—Cl1	−178.1 (2)	C16—C17—N1—C11	−129.4 (2)
C21—C22—C23—C24	−1.3 (6)	C18—C17—N1—C11	−3.3 (3)
Cl1—C22—C23—C24	178.4 (3)	C41A—C40—N2—C43	−26.4 (14)
C20—C19—C24—C23	0.1 (5)	C41—C40—N2—C43	11.5 (6)
C18—C19—C24—C23	179.5 (3)	C41A—C40—N2—C37	97.7 (14)
C22—C23—C24—C19	0.4 (5)	C41—C40—N2—C37	135.6 (6)
C13—C12—C25—O2	33.3 (4)	C42—C43—N2—C40	10.8 (4)
C11—C12—C25—O2	155.8 (3)	C44—C43—N2—C40	137.3 (3)
C18—C12—C25—O2	−88.0 (3)	C42—C43—N2—C37	−118.0 (3)
C13—C12—C25—O3	−147.2 (2)	C44—C43—N2—C37	8.4 (3)
C11—C12—C25—O3	−24.8 (3)	C35—C37—N2—C40	84.8 (3)
C18—C12—C25—O3	91.5 (3)	C38—C37—N2—C40	−155.3 (3)
C36—C27—C28—C29	0.4 (4)	C35—C37—N2—C43	−152.7 (2)
C27—C28—C29—C30	0.0 (5)	C38—C37—N2—C43	−32.8 (3)
C28—C29—C30—C31	0.9 (5)	C9—C8—O1—C13	−21.0 (3)
C29—C30—C31—C32	176.5 (3)	C7—C8—O1—C13	160.3 (2)
C29—C30—C31—C36	−2.3 (4)	C12—C13—O1—C8	50.0 (3)
C30—C31—C32—C33	−177.6 (3)	O2—C25—O3—C26	3.1 (5)
C36—C31—C32—C33	1.2 (4)	C12—C25—O3—C26	−176.4 (3)
C31—C32—C33—C34	−1.6 (4)	C35—C34—O4—C39	−14.4 (4)
C32—C33—C34—O4	178.6 (3)	C33—C34—O4—C39	165.8 (2)
C32—C33—C34—C35	−1.3 (4)	C38—C39—O4—C34	41.8 (3)
O4—C34—C35—C36	−175.5 (2)	O5—C51—O6—C52	−1.4 (5)
C33—C34—C35—C36	4.3 (4)	C38—C51—O6—C52	−177.7 (3)
O4—C34—C35—C37	4.3 (4)	C43—C42—C41—C40	35.3 (8)
C33—C34—C35—C37	−175.9 (2)	C41A—C42—C41—C40	−57.7 (9)
C28—C27—C36—C31	−1.7 (4)	C41A—C40—C41—C42	69.9 (11)
C28—C27—C36—C35	−179.8 (2)	N2—C40—C41—C42	−29.8 (9)
C32—C31—C36—C27	−176.2 (2)	N2—C40—C41A—C42	30.0 (19)
C30—C31—C36—C27	2.6 (4)	C41—C40—C41A—C42	−61.9 (14)
C32—C31—C36—C35	2.0 (4)	C41—C42—C41A—C40	72.3 (15)
C30—C31—C36—C35	−179.2 (2)	C43—C42—C41A—C40	−22.1 (18)

### Hydrogen-bond geometry (Å, °)

**Table:**

Cg5, Cg11 and Cg12 are the centroids of the C27–C31/C36, C1–C5/C10 and C5–C10 rings, respectively.

**Table:**

*D*—H···*A*	*D*—H	H···*A*	*D*···*A*	*D*—H···*A*
C11—H11···O3	0.98	2.39	2.773 (3)	103
C37—H37···O6	0.98	2.48	2.818 (3)	100
C14—H14A···Cg12^i^	0.97	3.00	3.951 (2)	168
C16—H16A···Cg11^i^	0.97	2.83	3.706 (2)	151
C21—H21···Cg11^ii^	0.93	2.85	3.649 (2)	144
C26—H26B···Cg5^iii^	0.96	2.83	3.555 (1)	133

Symmetry codes: (i) −*x*, *y*−1/2, −*z*+1/2; (ii) −*x*, −*y*+1, −*z*; (iii) −*x*+1, *y*+1/2, −*z*+1/2.
